# Characterization of melanin and optimal conditions for pigment production by an endophytic fungus, *Spissiomyces endophytica* SDBR-CMU319

**DOI:** 10.1371/journal.pone.0222187

**Published:** 2019-09-09

**Authors:** Nakarin Suwannarach, Jaturong Kumla, Bunta Watanabe, Kenji Matsui, Saisamorn Lumyong

**Affiliations:** 1 Department of Biology, Faculty of Science, Chiang Mai University, Chiang Mai, Thailand; 2 Center of Excellence in Microbial Diversity and Sustainable Utilization, Chiang Mai University, Chiang Mai, Thailand; 3 Institute for Chemical Research, Kyoto University, Kyoto, Japan; 4 Graduate School of Sciences and Technology for Innovation (Agriculture), Yamaguchi University, Yamaguchi, Japan; 5 Academy of Science, The Royal Society of Thailand, Bangkok, Thailand; Leibniz-Institut fur Naturstoff-Forschung und Infektionsbiologie eV Hans-Knoll-Institut, GERMANY

## Abstract

Melanin is a natural pigment that is produced by filamentous fungi. In this study, the endophytic species, *Spissiomyces endophytica* (strain SDBR-CMU319), produced a brown-black pigment in the mycelia. Consequently, the pigment was extracted from the dried fungal biomass. This was followed by pigment purification, characterization and identification. Physical and chemical characteristics of the pigment showed acid precipitation, alkali solubilization, decolorization with oxidizing agents, and insolubility in most organic solvents and water. The pigment was confirmed as melanin based on ultraviolet-visible spectroscopy, Fourier-transform infrared, and electron paramagnetic resonance spectra analyses. The analyses of the elemental composition indicated that the pigment possessed a low percentage of nitrogen, and therefore, was not 3,4-dihydroxyphenylalanine melanin. Inhibition studies involving specific inhibitors, both tricyclazole and phthalide, and suggest that fungal melanin could be synthesized through the 1,8-dihydroxynaphthalene pathway. The optimum conditions for fungal pigment production from this species were investigated. The highest fungal pigment yield was observed in glucose yeast extract peptone medium at an initial pH value of 6.0 and at 25°C over three weeks of cultivation. This is the first report on the production and characterization of melanin obtained from the genus *Spissiomyces*.

## Introduction

Interest in natural pigments derived from microorganisms continues to increase and many research efforts have been made to replace synthetic pigments with natural pigments [1, 2]. Hence, consumer concern has increased regarding the potential long-term toxicity of synthetic pigments in food processing, cosmetics, pharmaceuticals and the textile industries due to their carcinogenicity, hyperallergenicity and other potential toxicological problems [3, 4]. Microbial pigments are advantageous in terms of their high availability, stability and yield, low residues and easy harvest ability [5, 6]. Microorganisms including algae, bacteria, fungi and protozoa are recognized as potential sources for various pigments, e.g. carotenoids, flavins, melamins, quinines, and more specifically monascin, phycocyanin or indigo [7, 8]. Many microbial pigments not only act as coloring agents, but also possess antioxidant, anti-inflammation and antimicrobial activities [5, 6, 8]. The selection of both an appropriate strain and fermentation process, as well as the selection of suitable media or substrates, are needed to significantly improve pigment production yields [9, 10].

Melanin, an insoluble and non-digestible dark brown to black pigment with a complex molecular structure, is generated by the polymerization of indolic and phenolic compounds and is widely distributed in animals, plants, and microorganisms [11–13]. Melanin possesses broad biological activities including; antioxidant, radioprotective, thermoregulative, chemoprotective, antitumor, antiviral, antimicrobial, immunostimulating and anti-inflammatory properties [12–14]. Several microorganisms (bacteria and fungi) produce melanin for their virulence in host associations and act against environmental stresses, e.g. ultraviolet ray, solar radiation, oxidant-mediated damages, temperature extremes, hydrolytic enzymes, heavy metal toxicity and antimicrobial drugs [15–17]. Microbial melanin is widely used in cosmetics, photo protective creams, the manufacturing of eyeglasses and the immobilization of radioactive waste [12, 18, 19]. Two major melanin synthesis pathways, 3,4-dihydroxyphenylalanine (DOPA) and 1,8-dihydroxynaphthalene (DHN), are found in fungi [12, 16, 20]. Many filamentous fungi in the genera *Alternaria*, *Armillaria*, *Aspergillus*, *Auricularia*, *Cladosporium*, *Epicoccum*, *Eurotium*, *Magnapothe*, *Ochroconis*, *Penicillium*, *Phomopsis*, *Sporothri*, *Stachybotrys* and *Wangiella* have been reported as melanin producers [21–30]. In addition, some yeast species e.g. *Aureobasidium pullulans*, *Candida albicans*, *Cryptococcus neoformans*, *Hormoconis resinae* and *Kluyveromyces marxianus* have been reported for melanin production [16, 31–33]. In this study, a brown-black pigment produced from an endophytic fungus, *Spissiomyces endophytica* SDBR-CMU319 [34], was extracted from dried fungal biomass. The physical and chemical properties of fungal pigment were investigated with ultraviolet-visible absorption spectrometry, Fourier-transform infrared (FT-IR) spectroscopy, electron paramagnetic resonance (EPR), and elemental composition analyses in comparison with the synthetic DOPA-melanin standard. The effect of specific inhibitors on the melanin synthesis pathway was used to determine the melanin synthesis pathway in this fungus. Moreover, the optimal conditions (culture medium, pH value and temperature) for fungal pigment production were determined.

## Materials and methods

### Fungal strain

**Fig 1:**
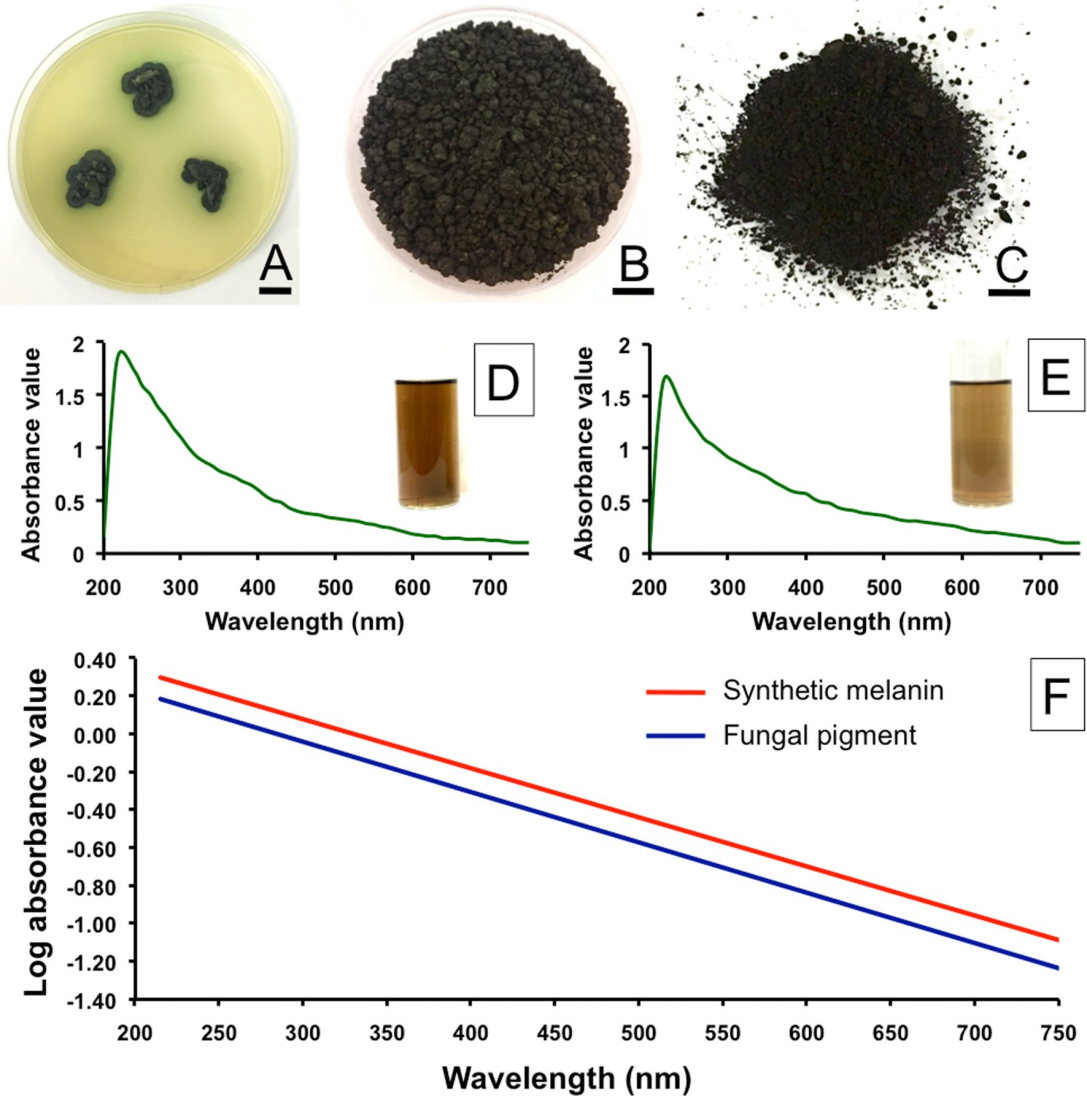
Colonies of *Spissiomyces endophytica* SDBR-CMU319 on potato dextrose agar at 25°C for three weeks (A). Dried fungal biomass of *Spissiomyces endophytica* SDBR-CMU319 after cultivation in potato dextrose broth at 25°C for three weeks (B). Pellets of fungal pigment after extraction and purification (C). UV and visible spectra of a synthetic DOPA-melanin standard (D) and the extracted fungal pigment (E). Linear plots with negative slopes of DOPA-melanin standard and the extracted fungal pigment. Bar A and B = 10 mm, C = 5 mm.

*Spissiomyces endophytica* SDBR-CMU319, an endophytic fungus isolated from *Balanophora fungosa* J.R. Forst. G. Forst. was stored in a 15% glycerol solution at -20°C at the Sustainable Development of Biological Resources Laboratory, Faculty of Science, Chiang Mai University, Chiang Mai, Thailand. From this stock culture, new cultures were produced by transferring *S*. *endophytica* agar plugs to the center of potato dextrose agar (PDA; CONDA, Spain) plates an incubated at 25°C in the darkness (Fig 1A).

### Cultivation of fungal pigment production

Ten fungal mycelial plugs (5 mm in diameter) obtained from the periphery of the growing colony on PDA at 25°C for three weeks were transferred into 250 mL of potato dextrose broth (PDB; CONDA, Spain), pH 6.0 in each 500-mL Erlenmeyer flask after being autoclaved at 121°C for 15 min. Cultivation was performed in the dark at 25°C with shaking at 125 rpm on a reciprocal shaker for three weeks. After incubation, the cultures were centrifuged at 11000 rpm for 15 min to harvest the fungal mycelia. The fungal mycelia were dried at 60°C for 48 h and thereafter, it was chilled down in desiccators for 20 min before being weighed and kept at 4°C in the darkness.

### Extraction and purification of fungal pigment

Extraction and purification of pigment from a dried fungal biomass was performed as a described method by De la Rosa et al. [27] and Rajagopal et al. [29] with some modifications. Fungal pigment derived from 1 g of dried fungal biomass was dissolved in 5 ml of 1 mol/L KOH, allowed to stand for 48 h and autoclaved (20 min at 121°C). Then, mixture was centrifuged at 5000 rpm and the resulting supernatant was acidified with 2 mol/L HCl to pH 2.5. Next, centrifugation at 5000 rpm for 5 min was performed to collect the precipitate, washed thrice with deionized water, dialysed and dried at 60°C for 48 h. Pellet was washed with chloroform, ethyl acetate and ethanol. This pellet (fungal pigment) was kept at -20°C until future use.

### Characterization of fungal pigment

The physical and chemical properties of pigment were determined following previous studies [25, 29, 35]. The solubility of extracted fungal pigment and synthetic DOPA-melanin standard (Sigma, USA) was tested with distilled water, 1 mol/L KOH, 1 mol/L NaOH, 100 mmol/L borate buffer (pH 8.0), 1 mol/L NaCl, methanol, absolute ethanol, acetone, acetonitrile, benzene, 1-butanol, ethyl acetate, chloroform, petroleum ether and 2-propanol. Precipitation in 1 mol/L HCl and 1% (w/v) FeCl_3_ were determined. Reactions with oxidizing agents (30% hydrogen peroxide and 10% sodium hypochlorite solutions) were determined.

### Detection and quantification of fungal pigment

#### Spectroscopic analysis

The obtained fungal pigment after purification (0.5 mg) was dissolved in 10 ml of 1 mol/L KOH following the method described by Rajagopal et al. [29]. The UV–visible absorption spectrum of the fungal was scanned in the wavelength range of 200–750 nm with a UV–visible spectrophotometer (BOEGO spectrophotometer model S-220 UV/VIS, Germany) by comparing a synthetic DOPA-melanin standard. The 1 mol/L KOH solution was used as reference blank. The maximum level of absorbance (λ_max_) of fungal pigment and synthetic DOPA-melanin standard were recorded.

#### Fourier-transform infrared resonance (FT-IR) analysis

FT-IR analysis was performed at the Center for Instrumental Analysis, Yamaguchi University, Yamaguchi, Japan. The purified fungal pigment and synthetic DOPA-melanin standard and were ground with IR grade potassium bromide and processed for FT-IR. The samples were pressed into disks under vacuum using a KBr press. The FT-IR spectra in the KBr discs were recorded on a Thermo Fisher Scientific Nicolet iS10 FT-IR Spectrometer (Thermo Fisher Scientific, USA). The spectra were read at a resolution of 4 cm^−1^ in the wave number region of 500–4000 cm^−1^.

#### Electron paramagnetic resonance (EPR) analysis

EPR spectra were taken at the Center for Instrumental Analysis, Yamaguchi University, Yamaguchi, Japan. The EPR spectra of the fungal pigment and synthetic DOPA-melanin standard were recorded in a solid state at 25°C in 4 mm quartz tubes on a Bruker ELEXSYS E500 spectrophotometer (Bruker Instruments Inc., USA). The various instrumental parameters of EPR were set at 100 kHz modulation frequency, 1.0 G modulation amplitude, 0.64 mW microwave power, 9.84 GHZ and 20.97 s scan time.

#### Elemental analysis

The elemental contents of fungal pigment and synthetic DOPA-melanin were determined by a Thermo Scientific FLASH 2000 Organic Elemental Analyzer (Thermo Fisher Scientific, USA).

### Determination of fungal melanin synthesis pathway using inhibitors

Melanin synthesis pathway was characterized by studying the effects of the inhibitors following the method described by previous studies [24, 25, 29] with some modifications. Twenty-five mL of PDB pH 6.0 were added in each 150-mL Erlenmeyer flask. After autoclaved, kojic acid (Sigma, USA) and tropolone (Tokyo Chemical Industry Co. Ltd., Japan) that inhibit DOPA pathway, and tricyclazole (Tokyo Chemical Industry Co. Ltd., Japan) and phthalide (Tokyo Chemical Industry Co. Ltd., Japan) that inhibit DHN melanin pathway were added into PDB at the final concentration 50 μg/ml in each flask. The media were inoculated with three fungal mycelial plugs (5 mm in diameter) obtained from the periphery of the growing colony on PDA at 25°C for three weeks and shaking at 25°C in the darkness for three weeks. A fungal growth and pigmentation were observed. Three replications were performed for each treatment.

### Optimization of fungal pigment production

#### Fungal cultivation

Three fungal mycelial plugs (5 mm in diameter) obtained from the periphery of the growing colony on PDA at 25°C for three weeks were transferred into 25 mL of liquid medium in each 125-mL Erlenmeyer flask after being autoclaved at 121°C for 15 min. Cultivation was performed in the dark at 25°C with shaking at 125 rpm on a reciprocal shaker. After incubation, the cultures were centrifuged at 11000 rpm for 15 min to harvest the fungal mycelia. The fungal mycelia were harvested and dried. The pigment was extracted as described above. Pigment yield was estimated following the method described by Wang et al. [36] in which the wavelength of its absorbance maxima was expressed in absorbance unit (AU). Five replicates were performed for each treatment.

#### Effect of culture liquid medium

Five different liquid media were used in this experiment; PDB, Czapek Dox broth (CDB; Signma-Aldrich, USA), glucose yeast extract peptone medium (GYPM; glucose 10 g, yeast extract 2 g, peptone 1 g), malt extract medium (ME; malt extract 20 g) and oatmeal medium (OM; Difco, USA). In all liquid media, the volume was adjusted to 1000 mL with distilled water and the pH was adjusted to 6.0 using 1 mol/L HCl and 1 mol/L NaOH. After inoculation, the culture media were incubated in darkness at 25°C for three weeks with shaking. The liquid medium that presented the highest yield of pigment was selected for further experiments.

#### Effect of initial pH and temperature

The initial pH of the selected suitable liquid medium that had been obtained from previous experiments was adjusted from 3.0 to 9.0 before being autoclaving. After inoculation, the culture media were incubated in darkness at 25°C for three weeks with shaking. The initial pH value that presented the highest yield of pigment was selected for further experiments. The fungus was grown in the darkness at 15, 20, 25, 30, 35 and 40°C for three weeks with shaking to find out the optimal temperature for pigment production.

### Statistical analysis

Statistical analyses were carried out by one-way analysis of variance (ANOVA) using SPSS program version 16.0 for Windows. Tukey’s range test was used to determine significant differences (*P*<0.05) between the mean values of each treatment.

## Results

### Detection and quantification of pigment production by *Spissiomyces endophytica* SDBR-CMU319

Extraction of pigments produced from *S*. *endophytica* was achieved from dried fungal biomass (Fig 1B). The purified pigment appeared as a dark brown in color (Fig 1C). Extraction and purification of the pigment produced yields of 315.20 ± 13.57 mg per gram of dried fungal biomass with five replications.

### Characterization of *Spissiomyces endophytica* SDBR-CMU319 pigment

The physical and chemical properties of *S*. *endophytica* pigment and synthetic DOPA-melanin standard are shown in Table 1. The fungal pigment and synthetic DOPA-melanin standard were insoluble in distilled water, 1 mol/L NaCl, methanol, absolute ethanol, acetone, acetonitrile, benzene, 1-butanol, ethyl acetate, chloroform, petroleum ether and 2-propanol. However, the fungal pigment and the synthetic DOPA-melanin standard revealed solubility in 1 mol/L KOH, 1 mol/L NaOH and 100 mmol/L borate buffer. Both fungal pigment and the synthetic DOPA-melanin standard displayed precipitation in 1 mol/L HCl and 1% FeCl_3_ solutions. Both the fungal pigment and synthetic DOPA-melanin were positive for decolorization by 30% hydrogen peroxide and 10% sodium hypochlorite solutions. The chemical properties of the *S*. *endophytica* pigment were almost similar with those of the synthetic DOPA-melanin standard.

**Table 1:** Physical and chemical properties of fungal pigment and synthetic DOPA-melanin.

No.	Test	Result	
		Fungal pigment	Synthetic DOPA-melanin
1.	Color observation	Blackish brown	Blackish brown
2.	Solubility test		
	a) Distilled water	Insoluble	Insoluble
	b) 1 mol/L KOH	Soluble	Soluble
	c) 1 mol/L NaOH	Soluble	Soluble
	d) 100 mmol/L borate buffer	Soluble	Soluble
	e) 1 mol/L NaCl	Insoluble	Insoluble
	f) Methanol	Insoluble	Insoluble
	g) Absolute ethanol	Insoluble	Insoluble
	h) Acetone	Insoluble	Insoluble
	i) Acetonitrile	Insoluble	Insoluble
	j) Benzene	Insoluble	Insoluble
	k) 1-Butanol	Insoluble	Insoluble
	l) Ethyl acetate	Insoluble	Insoluble
	m) Chloroform	Insoluble	Insoluble
	n) Petroleum ether	Insoluble	Insoluble
	o) 2-Propanol	Insoluble	Insoluble
3.	Precipitation test		
	a) 3 mol/L HCl	Readily precipitate	Readily precipitate
	b)1% FeCl_3_	Brown precipitate	Brown precipitate
4.	Reaction with oxidizing agent		
	a) 30% hydrogen peroxide	Decolorized	Decolorized
	b) 10% sodium hypochlorite	Decolorized	Decolorized

### Detection and quantification of fungal pigment

#### Spectroscopic analysis

The wavelength of maximum absorbance was scanned at a range of 200 to 750 nm. The wavelength of maximum absorbance of the extracted fungal pigment and synthetic DOPA-melanin standard was observed at 215 nm (Fig 1D and 1E). In this study, the graph of log absorbance against the wavelength of the fungal pigment and synthetic DOPA-melanin were similar and presented a straight line with a negative slope at -0.0027 and -0.0026, respectively (Fig 1F). Therefore, the pigment produced from *S*. *endophytica* was verified as melanin.

#### FT-IR analysis

**Fig 2:**
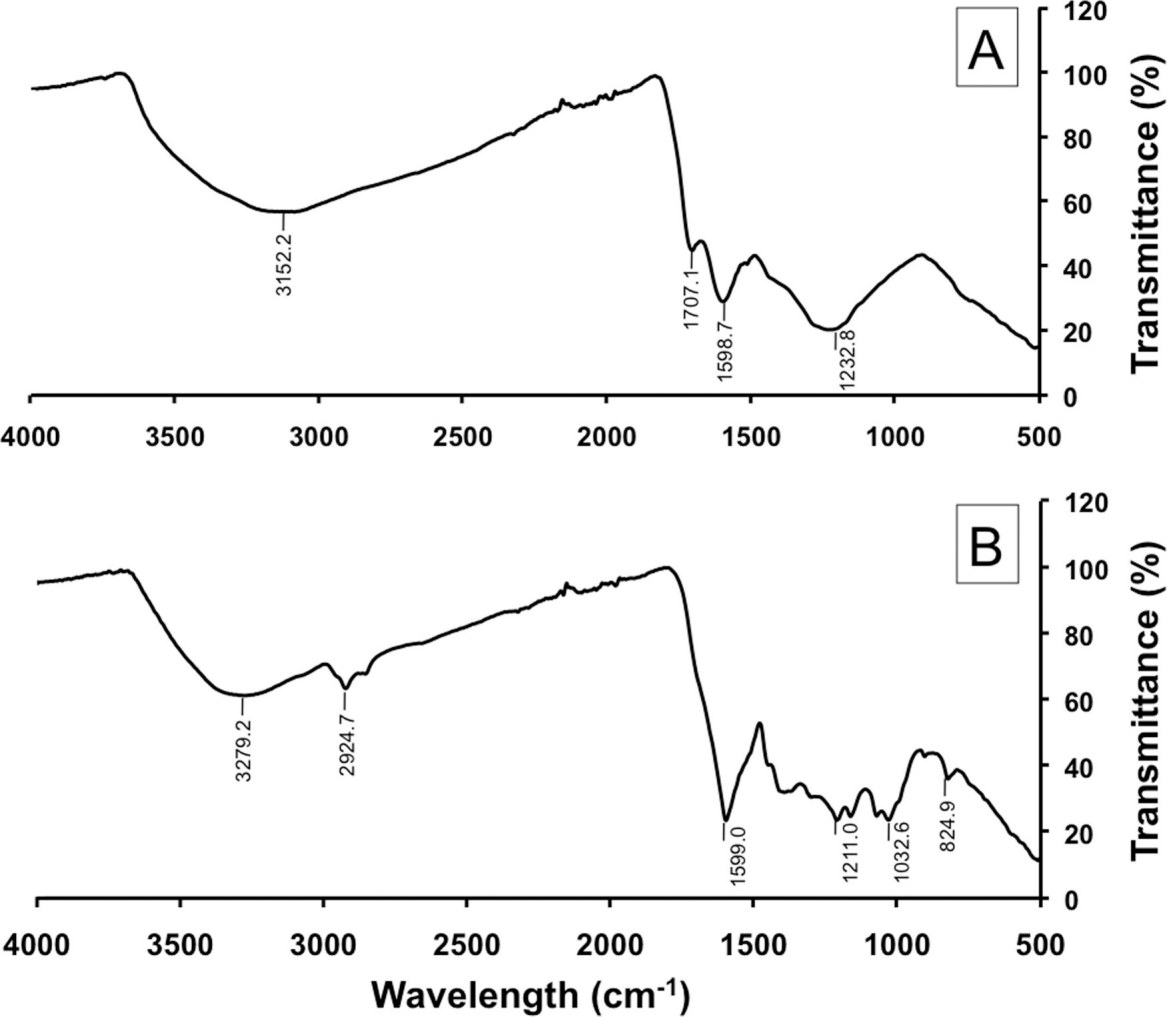
Fourier-transform infrared resonance spectra of a synthetic DOPA-melanin standard (A) and the extracted fungal pigment (B).

The FT-IR spectra were analyzed to confirm that the extracted fungal pigment was melanin. The FT-IR spectra of the extracted fungal pigment and the synthetic DOPA-melanin standard are shown in Fig 2. Both spectra revealed broad absorption peaks at 3300−3000 cm^-1^, which can be attributed to the stretching vibrations of the OH groups [36, 37]. An absorption peak of about 1707.1 cm^-1^ in the spectra of the synthetic DOPA-melanin standard was assigned to COOH stretching [38]. The spectra of the extracted fungal pigment and the synthetic DOPA-melanin standard exhibited a strong level of absorption at 1650−1500 cm^-1^ with assigned aromatic ring C = C stretching [36]. The absorption peak in both spectra were recorded at 1210−1230 cm^-1^ and assigned to C−OH stretching and OH deformation of the alcoholic [37–39]. In addition, the absorption peak recorded at 824.9 indicated O−H bending [40]. The absorption peak recorded at 2924.7 and 1032.6 cm^-1^ in the spectra of fungal pigment indicated the saturated carbon and C−O stretching of polysaccharides that may be contaminated by the cell wall carbohydrate [38, 41, 42].

#### EPR analysis

**Fig 3:**
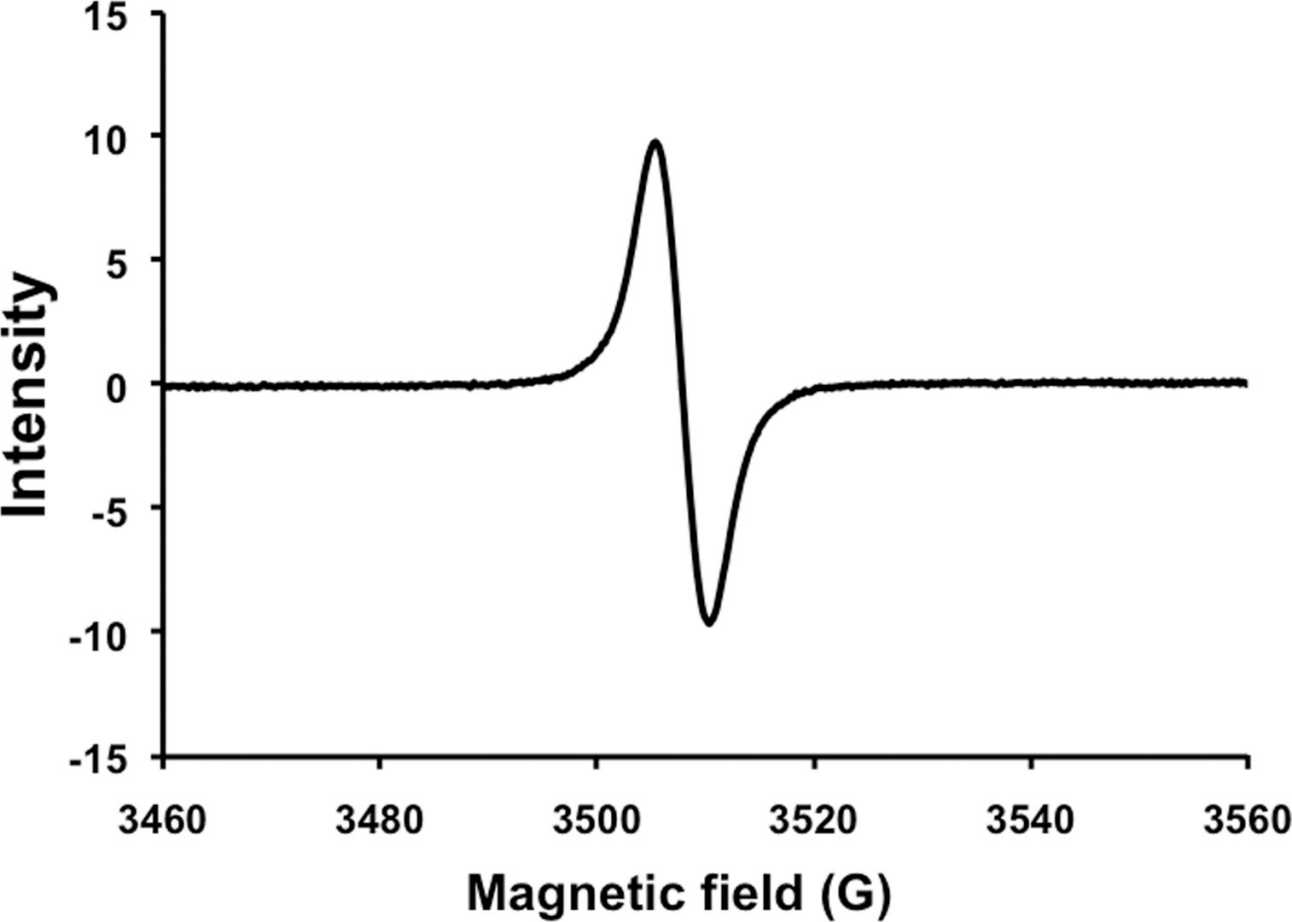
Electron paramagnetic resonance spectra of the extracted fungal pigment.

Due to the presence of organic free radicals, the characteristic behavior of EPR spectroscopy is another diagnostic feature of melanin [43, 44]. In this study, EPR revealed that pigment particles obtained from the fungal cells of *S*. *endophytica* contained a stable free radical compound (Fig 3). The EPR of the fungal pigment was similar to the EPR signal of the synthetic DOPA-melanin with a G value equal to 3510.

#### Elemental analysis

The percentage contents of C, H, N, O and S in the fungal pigment and synthetic DOPA-melanin are presented in Table 2. An elemental analysis of the fungal pigment showed 52.69% C, 4.69% H and 0.47% N contents. The synthetic DOPA-melanin revealed 46.50% C, 3.14% H and 5.95% N contents. Notably, the fungal pigment had a lower %N value than the synthetic DOPA-melanin.

**Table 2:** Element composition of fungal pigment and synthetic DOPA-melanin.

Sample	Element composition (%)^a^				
	Carbon (C)	Hydrogen (H)	Nitrogen (N)	Sulfur (S)	Oxygen (O)^b^
Synthetic DOPA-melanin	46.50	3.14	5.95	0.00	44.01
Fungal pigment	52.69	4.69	0.47	0.00	42.15

^a^ Data were means of duplicate.

^b^The content of oxygen element was calculated from the equation: O% = 100%−C%−H%−N%−S%

### Determination of fungal melanin synthesis pathway by *Spissiomyces endophytica* SDBR-CMU319 using inhibitors

**Fig 4:**
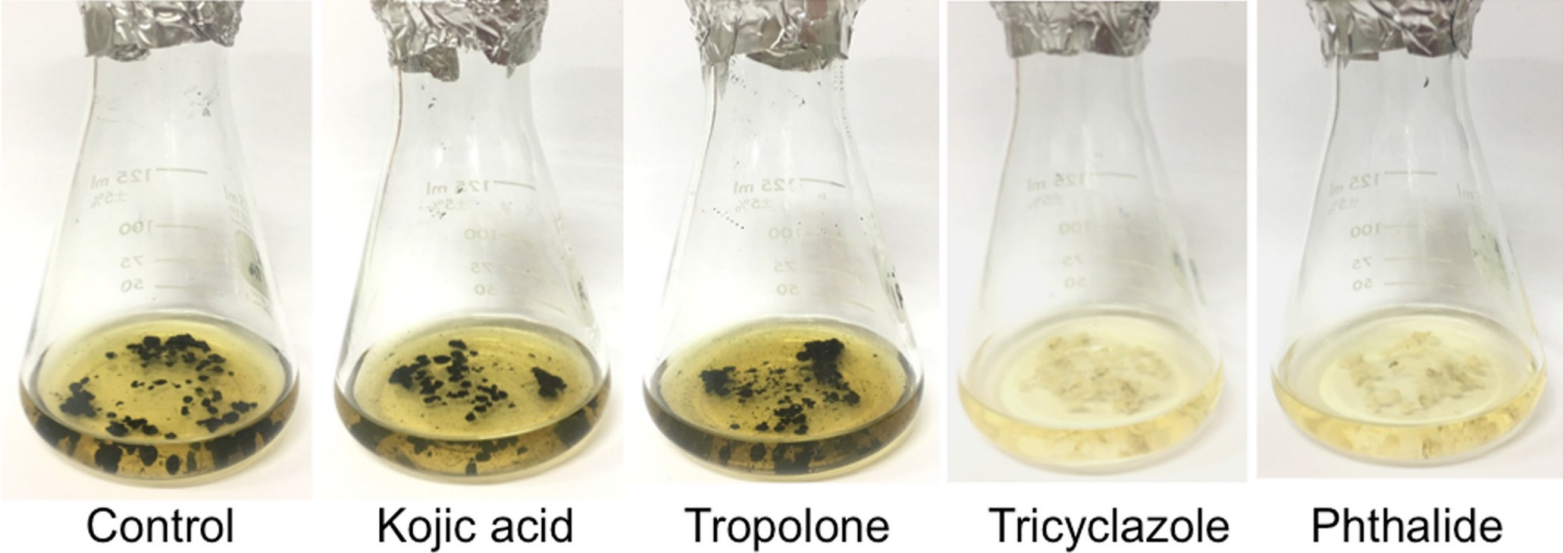
Pigmentation of *Spissiomyces endophytica* SDBR-CMU319 on potato dextrose broth in the absence of melanin biosynthesis inhibitor (control), present of biosynthesis inhibitors of DOPA-melanin (kojic acid and tropolone) and present of biosynthesis inhibitors of DHN-melanin (tricyclazole and phthalide).

The fungi were grown in the presence of several inhibitors, such as kojic acid, tropolone, tricyclazole, or phtalide. After three weeks, we found that tricyclazole and phtalide effectively suppressed formation of melanin (Fig 4).

### Optimization of fungal pigment production by *Spissiomyces endophytica* SDBR-CMU319

**Fig 5:**
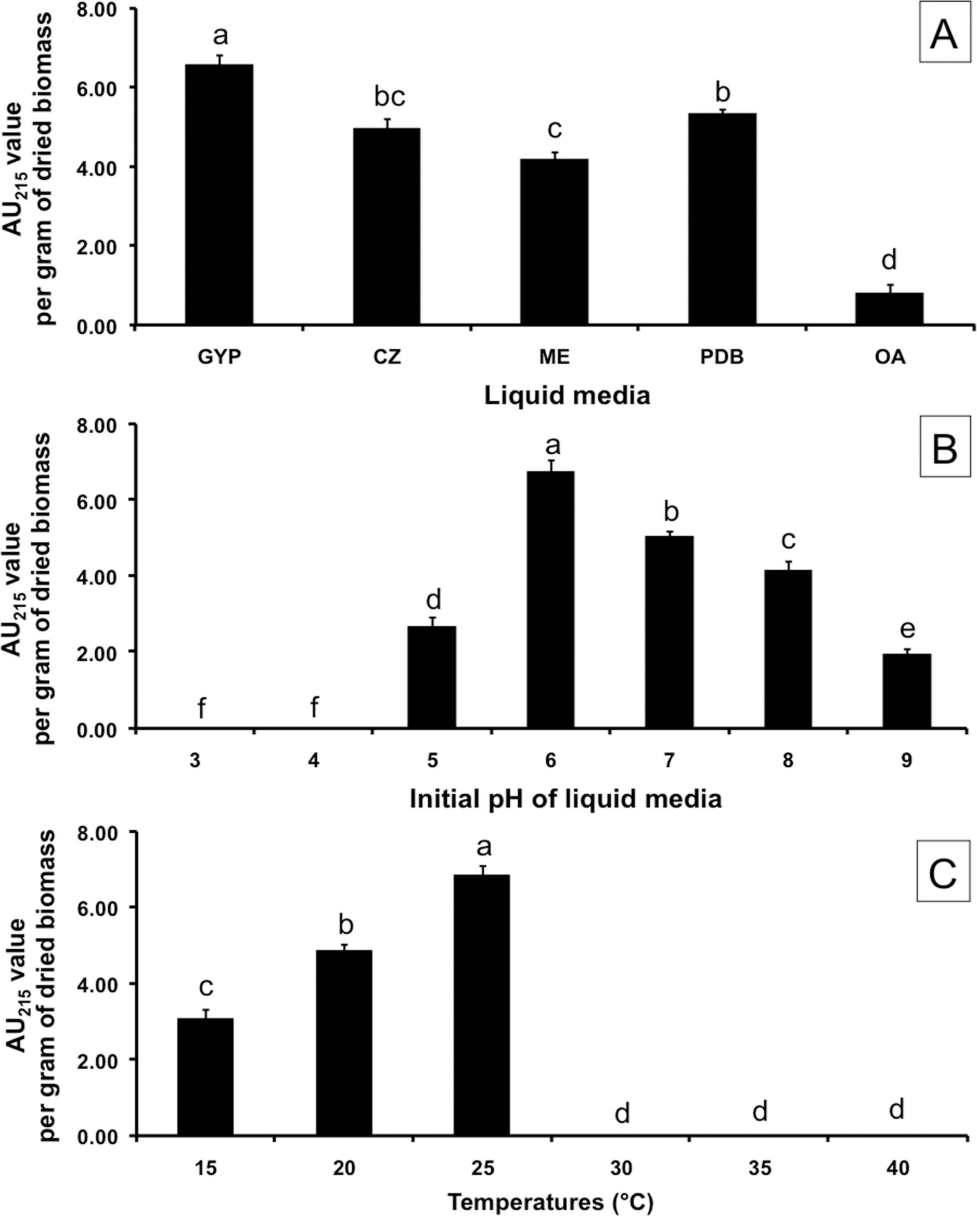
Effects of liquid media (A), initial pH of liquid medium (B), temperatures (C) on fungal melanin production by *Spissiomyces endophytica* SDBR-CMU319. The results are means of five replicates ± SD. Different letters above each bar in the same parameter indicate the significant difference (*P<*0.05). GYP = glucose yeast extract peptone medium, CZ = Czapek Dox broth, ME = malt extract medium, PDB = potato dextrose broth and OA = oatmeal medium.

The results showed that the wavelength of maximum absorbance of fungal pigment was observed at 215 nm; therefore, indication of the total pigment yield was estimated at this wavelength. Values were normalized to AU_215_ per gram of dried fungal biomass. Fungal pigment production levels of *S*. *endophytica* in various liquid media are shown in Fig 5A. The results indicate that the highest significant fungal pigment yield (6.56 ± 0.25 AU_215_ per gram of dried fungal biomass) was observed in the GYP medium followed by PDB (5.32 ± 0.13 AU_215_ per gram of dried fungal biomass) in the CZ medium (4.95 ± 0.25 AU_215_ per gram of dried fungal biomass), while the lowest fungal pigment yield was found in the OM medium (0.78 ± 0.23 AU_215_ per gram of dried fungal biomass). Our results indicate that the initial pH value of the liquid medium affected fungal pigment production (Fig 5B). The highest significant fungal pigment yield (6.72 ± 0.32 AU_215_ per gram of dried fungal biomass) was found in the GYP medium at a pH of 6.0, followed by a pH of 7.0 (5.02 ± 0.15 AU_215_ per gram of dried fungal biomass) and a pH of 8.0 (4.13 ± 0.25 AU_215_ per gram of dried fungal biomass). Notably, the fungus could not grow at pH values of 3.0 and 4.0. Temperature affected fungal pigment production (Fig 5C). The highest fungal pigment yield (6.84 ± 0.24 AU_215_ per gram of dried fungal biomass) was observed at 25°C, which was the optimum temperature. However, the fungus could not grow at 30, 35 and 40°C.

## Discussion

Filamentous fungi are natural sources for the production of pigments [13, 15, 16, 33]. In the present study, an endophytic fungus, *S*. *endophytica* (strain SDBR-CMU319), produced melanin pigment in fungal mycelia wherein the fungal melanin was extracted from the fungal biomass. The results were similar to those of previous studies which found that pure cultures of filamentous fungi e.g. *Alternaria alternata*, *Aspergillus flavus*, *A*. *fumigatus*, *A*. *nidulans*, *A*. *niger*, *A*. *sydowii*, *A*. *tamari*, *A*. *terreus*, *A*. *tubingensis*, *Epicoccum nigrum*, *Exophiala pisciphila*, *Magnapothe grisea*, *Ochroconis anomala*, *O*. *lascauxensis*, *Penicillium marneffei*, *Phyllosticta capitalensis* and *Stachybotry chartarum* could produce melanin after being cultured in both solid and liquid media. Notably, some fungal melanin were present in the fungal structures (e.g. cell wall, appressoria and spore) or secreted into the cultivated medium [16, 24–26, 28, 27, 45, 46]. However, the present study has provided the first report on melanin pigments being produced by *S*. *endophytica*. The primary identification of melanin commonly relies on the criteria of the physical and chemical properties. Our results show that the melanin pigment produced by *S*. *endophytica* was soluble in alkali solutions, insoluble in water and organic solvents and salt solutions, precipitated in HCl solution, decolorized in the presence of oxidizing agents and displayed positive reactions for polyphenols by producing flocculent brown precipitation with FeCl_3_. These characteristics were also exhibited in the synthetic melanin and are common to various microbial melanin’s as have been described in previous studies [25, 27, 29, 35, 45, 47].

To confirm that the fungal pigment was melanin, UV, FT-IR, and EPR spectra analyses were used. In the present study, the UV-visible absorbance spectrum of the fungal pigment produced from *S*. *endophytica* showed a strong absorbance in the UV region. The maximum absorbance was observed at 215 nm and decreased towards the visible region. These results were similar to those of previous studies which found that the highest level of absorbance of the melanin produced by various fungi were in the UV region ranging from 200−300 nm and decreased toward the visible region [16, 25, 26, 29, 45, 46, 48, 49]. The fungal pigment solution in this study displayed a log of optical density when plotted against wavelength and produced a linear curve with negative slopes. Similarly, the characteristic straight lines with a negative slope were obtained for the melanin produced by fungi and the synthetic melanin [12, 21, 25, 26, 29, 49]. The IR spectrum of the fungal pigment showed a broad absorption level and revealed the presence of hydrogen bonding of the OH group and aromatic ring C = C stretching. These characteristic properties of the IR spectrum of this pigment were similar to those of previous reports on the properties of fungal melanin, as well as with the properties of synthetic melanin [12, 25, 26, 29, 50]. EPR spectroscopy is known to be a particularly effective method for studying fungal melanin since this melanin uniquely contains a stable population of organic free radicals [21, 43, 51–53]. In the present study, the fungal pigment contained a stable free radical compound, and EPR signal of the fungal pigment pattern appeared to have a similar signal of the fungal melanin and the synthetic melanin standard that had been reported [21, 25, 26, 29, 45, 50].

Generally, melanins synthesized from the DOPA-pathway are divided into eumelnin and pheomelanin. Eumelanin contained 5.1−9% nitrogen and 0−1% sulfur, while pheomelanin contained 8−11% nitrogen and 9−12% sulfur [12, 35, 45, 54]. On the other hand, melanins synthesized from the DHN-pathway were present only with a trace of nitrogen [12, 13, 16]. Our study also carried out an elemental composition analysis of the pigment extracted from *S*. *endophytica*. The obtained fungal pigment revealed a low percentage of nitrogen (0.47%), which differed from the findings of previous studies on DOPA-melanin that had been synthesized from fungi. Notably, previous studies on DOPA-melanin synthesized from fungi, whose percentage of nitrogen ranged from 3 to 6.7% [12, 22, 35, 41, 45, 54, 55]. Therefore, it was determined that the pigment extracted from *S*. *endophytica* SDBR-CMU319 was not a DOPA-melanin, but probably a DHN-melanin. The nitrogen present in the fungal melanin in our study suggested that it may be an unpurified fungal pigment or may occur as a small degree of contamination of a nitrogen-containing compound that was attached to the melanin [12, 16, 43].

The compounds that specifically inhibit the biosynthesis pathway of the DOPA- and DHN-melanin were tested in microorganisms. Tricyclazole and phthalide inhibit the enzymatic reduction of two hydroxynapthalene compounds to scytalone and vermelone, which are the intermediates in the melanin produced from the DHN-pathway. However, kojic acid and tropolone inhibited tyrosinase, an enzyme responsible for the production of DOPA-melanin [11, 25, 56]. In the present study, the presence of tricyclazole and phthalide revealed the lack of pigmentation of *S*. *endophytica*, an ascomycetous fungus [34], which suggested that the melanin pigment was produced by this fungus from the DHN-pathway. This is similar to previous studies which found that sexual and asexual ascomycetes predominantly produced melanin via the DHN-pathway, but basidiomycetes produced melanin via the DOPA-pathway [12, 23, 24, 35, 47, 48]. However, some ascomycete species, e.g. *A*. *nidulans*, *A*. *niger*, *A*. *tamari*, *A*. *flavus*, *Cladosporium resinae*, *Epicoccum nigrum*, *Hendendersonula toruloidea*, *Eurotium echinulatum*, *Humicola grisea* and *Hypoxylon archeri*, produced melanin via the DOPA-pathway [21, 23, 25, 26, 45, 51, 53, 57, 58]. In addition, Pal et al. [25] found that the amount and type of the melanin synthesis pathway in *Aspergillus* differed from the other fungal species, and Sapmak et al. [59] found that *Talaromyces marneffei* (basionym: *Penicillium marneffei*) could synthesize both DOPA- and DHN-melanin depending on the growth conditions and the supply of relevant precursors.

Our results reveal that the cultivated liquid medium, initial pH medium and temperature all affected fungal melanin production. The optimum conditions for the highest yield of fungal melanin production from *S*. *endophytica* was obtained at three weeks of cultivation in the GYP medium, at a pH of 6.0 and at an incubation temperature of 25°C. This result was supported by the findings of several previous studies which found that fungal melanin production yields were greatly influenced by the relevant cultivation conditions (e.g. cultivation medium, temperature, pH, aeration and type of fermentation), and that the optimum conditions for melanin production were not necessarily homologous for the fungal species and strains [19, 20, 33, 60]. Zhang et al. [50] and Raman et al. [46] reported that the optimization of the media composition could enhance the production of melanin by a submerged culture of *Auricularia auricular* and *A*. *fumigatus*, respectively. Moreover, the determination of the optimum conditions for microbial melanin production could result in an increase in melanin yields for large-scale production [19, 47, 61].

## Conclusion

*Spissiomyces endophytica* SDBR-CMU319 produced pigments that were characterized as melanin on the basis of their physicochemical properties. Based on the elemental composition patterns of fungal melanin and the specific inhibitors of the melanin synthesis pathway, we have concluded that this fungus could produce melanin via the DHN-pathway. In addition, the highest fungal pigment yield was observed in the glucose yeast extract peptone medium at a pH value of 6.0 and at a temperature of 25°C over three weeks of cultivation. Further studies on melanin obtained from this fungus are required to evaluate its toxicity for the purposes of replacing the commercial pigments that are currently being used, and for the purposes of developing melanin for large scale commercial production.
